# Impact of Acute Coronary Syndrome on Clinical Outcomes in Patients With Thrombotic Thrombocytopenic Purpura

**DOI:** 10.7759/cureus.35878

**Published:** 2023-03-07

**Authors:** Fouad Khalil, Mohammad Ali, Moataz Ellithi

**Affiliations:** 1 Internal Medicine, University of South Dakota Sanford School of Medicine, Sioux Falls, USA; 2 Hematology and Oncology, University of Nebraska Medical Center, Sioux Falls, USA

**Keywords:** acute coronary syndrome, myocardial ischemia, hemolytic, anemia, thrombocytopenia, thrombotic microangiopathies

## Abstract

Thrombotic thrombocytopenic purpura (TTP) is a life-threatening clinical syndrome characterized by microangiopathy and a variable degree of end-organ ischemic damage. Cardiac involvement has been recognized as a major cause of mortality in these patients. In this study, we queried the National Inpatient Sample (NIS) for all patients hospitalized with thrombotic microangiopathy from 2002 to 2017, who also received plasma exchange (PLEX) during the same admission. A total of 6,214 patients with TTP were identified. We stratified patients based on whether or not they had acute coronary syndrome (ACS) during admission. ACS was documented in 6.3% of patients. Compared with patients without ACS, those with ACS were relatively older (odds ratio [OR], 1.03; 95% confidence interval [CI], 1.02-1.03) and had a relatively higher prevalence of heart failure (OR, 2.02; 95% CI, 1.53-2.67) and coronary artery disease (OR, 2.69; 95% CI, 2.03-3.57). Certain complications were more prevalent in the ACS group including acute cerebrovascular accident (OR, 3.33; 95% CI, 2.94-3.78), acute heart failure (OR, 1.91; 95% CI, 1.67-2.19), acute kidney injury (OR, 1.76; 95% CI, 1.59-1.95), cardiogenic shock (OR, 2.15; 95% CI, 1.72-2.69), and respiratory failure (OR, 1.48; 95% CI, 1.32-1.66). Despite wider utilization of therapeutic plasmapheresis and improved supportive management of patients with TTP, associated morbidity and mortality remain significant. We demonstrate from this large retrospective cohort that ACS is an independent predictor of higher morbidity and mortality in TTP patients.

## Introduction

Thrombotic thrombocytopenic purpura (TTP) is a rare and life-threatening clinical syndrome characterized by microangiopathic hemolytic anemia and thrombocytopenia with subsequent development of variable degrees of end-organ ischemic damage. It was first described by Eli Moschcowitz in 1925 [1]. More cases were subsequently reported in the following years and the classical pentad of fever, thrombocytopenia, microangiopathic hemolytic anemia, neurological symptoms, and renal insufficiency was first coined by Amorosi and Ultmann in their seminal paper in 1966. Nonetheless, more recent studies have found that the classic pentad was present in less than 10% of patients with TTP [2].

The relative deficiency of a specific von Willebrand factor-cleaving protease, ADAMTS13 (a disintegrin and metalloproteinase with a thrombospondin type 1 motif, member 13), is implicated as the inciting event that mediates subsequent activation of the coagulation cascade with the resultant endothelial damage and end-organ ischemia. ADAMTS13 deficiency is frequently acquired through the formation of ADAMTS13 autoantibodies but is less commonly inherited via mutations in the coding gene [3].

More wide utilization of therapeutic plasmapheresis in eligible patients and improved supportive care have led to significant improvement in the survival of TTP patients; nonetheless, the disease has a mortality of 10% with appropriate treatment and is associated with significant morbidity. For instance, cardiac involvement has been recognized as a major cause of mortality in these patients [4,5]. This was also supported by the autopsy data showing diffuse myocardial ischemia in the majority of patients with TTP [6,7]. Heart ischemia occurs in up to 25% of patients with TTP, ranging from isolated electrocardiographic abnormalities to myocardial ischemia and acute coronary syndrome (ACS) [5].

A summary of the study results was previously presented as a meeting abstract at the 2020 ASH Annual Scientific Meeting on December 6, 2020.

## Materials and methods

Data source

Data were obtained from the National Inpatient Sample (NIS) database from 2002 to 2017. The NIS is the largest publicly available all-payer inpatient care database in the US, maintained by the Agency for Healthcare Research and Quality as a part of the Healthcare Cost and Utilization Project (HCUP). The NIS contains discharge-level data from approximately 8 million hospital stays per year, which approximately represent 20% of all US discharges. Criteria used for stratified sampling of hospitals into the NIS include hospital ownership, patient volume, teaching status, urban or rural location, and geographic region.

Study population

The NIS database was queried for all hospitalizations with a primary diagnosis of TTP (ICD-9-CM code 4466 and ICD-10-CM code M3.11) from 2002 to 2017. Using ICD-9-CM (9972), (9971), and (9979) codes as well as ICD-10-CM (6A551Z3) and (6A550Z3) codes, we identified patients who received plasma exchange (PLEX) during the same hospital admission. We decided to include all patients who carried the primary diagnosis of thrombotic microangiopathy (TMA) and had received PLEX during their admission. We stratified patients based on whether or not they had ACS during the same hospitalization, defined as the presence of an ICD code for either ST-segment elevation myocardial infarction (STEMI), non-STEMI, or unstable angina (see Appendix).

Outcome measures

The primary outcome of this study was the incidence and clinical predictors of ACS. In-patient mortality and morbidity including acute kidney injury, incident dialysis, acute respiratory failure, and encephalopathy were used as secondary outcomes.

Patient characteristics

Baseline characteristics used included demographics (age, sex, and race), primary payer, hospital region, hospital size, chronic medical problems (smoking, alcohol or drug abuse, obesity, dyslipidemia, hypertension, diabetes mellitus, coronary artery disease, peripheral vascular disease, congestive heart failure, and chronic kidney disease), and whether they received an early PLEX treatment, defined as within 24 hours of the admission, or not. A list of ICD-9-CM and ICD-10-CM codes used to identify risk factors and other pertinent comorbidities is provided in the Appendix.

Statistical analysis

Baseline characteristics were compared using Pearson’s chi-squared test for categorical variables and Student’s t-test for continuous variables. Cumulative in-hospital mortality among TTP patients with and without ACS was characterized using a Kaplan-Meier plot, with the log-rank (Mantel-Cox) test used for comparison between the two groups. Statistical analysis was performed using IBM SPSS Statistics 26.0 (IBM Corporation, Armonk, NY). We used a two-sided P value of <0.05 to assess statistical significance. Categorical variables are expressed as percentages and continuous variables as mean ± SD. The odds ratio (OR) and 95% confidence interval (CI) are used to report the results of the regression analysis.

## Results

Characteristics of the study population

**Figure 1:**
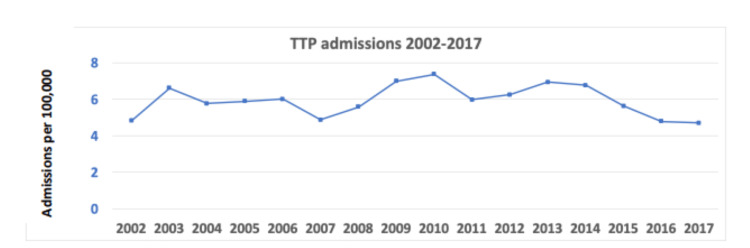
TTP annual admissions per every 100,000 admissions TTP, thrombotic thrombocytopenic purpura.

A total of 15,640 patients with the diagnosis of TTP were identified during the study period. Out of those, 6,214 patients had received PLEX treatment during their admission (39.7%). Due to the wide spectrum of TMA diseases, we decided to include only those who received PLEX to get a more specific subpopulation who were presumed to have TTP. The annual admission rate was ranging from five to seven admissions per 100,000 national admissions (Figure 1). Patients had a mean age of 47.8 years; 67% were females and 46.5% were Caucasian. Stratifying by geographic region, 24% were from the Northeast, 21% from the Midwest, 42% from the South, and 13% from the West. The most common primary payer was private insurance (42.7%).

Overall inpatient mortality was 9.1%. The most common complications reported included acute kidney injury (42.5%), followed by acute respiratory failure (14.9%), incident dialysis (14.3%), acute encephalopathy (7.7%), acute heart failure (7.3%), acute cerebrovascular accident (7.2%), and ACS (6.3%). 

Group comparison

Out of the 6,214 patients with TTP, 390 patients (6.3%) had ACS (Table 1). Compared with patients without ACS, those with ACS were relatively older and had a relatively higher prevalence of coronary artery disease, dyslipidemia, diabetes mellitus, essential hypertension, chronic kidney disease, and heart failure (Table 1). Patients with ACS had a three-fold higher in-hospital mortality (19.5% vs. 8.4%, P<0.001) and a longer mean hospital stay (19 days vs. 15 days, P<0.001) (Table 1). Moreover, the subgroup of patients with ACS was also found to have a higher incidence of acute kidney injury, incident dialysis, acute encephalopathy, acute respiratory failure, sepsis, acute congestive heart failure, cerebrovascular accidents, and cardiogenic shock.

**Table 1:** Baseline characteristics of patients admitted with TTP who developed ACS vs those who did not. TTP, thrombotic thrombocytopenic purpura; ACS, acute coronary syndrome.

Variable	TTP without ACS	TTP with ACS	P-value
	(n = 5,824)	(n = 390)	
Age	47.2 (16.4)	56.9 (16)	<0.001
Sex			0.025
Male	32.6%	38.2%	
Female	67.4%	61.8%	
Race			0.043
White	46.1%	51.9%	
Black	40.2%	37.0%	
Hispanic	8.4%	4.9%	
Others	4.2%	6.8%	
Median household income			0.024
Below 25^th^ percentile	31.7%	32.9%	
All other percentiles	68.3%	67.1%	
Hospital status			0.407
Rural	1.7%	0.8%	
Urban non-teaching	21.5%	21.6%	
Urban teaching	76.9%	77.6%	
Bed size			0.320
Small	7.2%	6.2%	
Medium	19.8%	22.7%	
Large	73.0%	71.1%	
Hospital region			0.005
Northeast	23.6%	28.5%	
Midwest	20.6%	24.6%	
South	42.5%	33.8%	
West	13.3%	13.1%	
Mean length of stay (days)	15	19	<0.001
In-hospital mortality	8.4%	19.5%	<0.001
Mean charges (US dollars)	161,798	224,166	<0.001
Mean number of diagnosis	11 (6)	15 (6)	<0.001
Acute kidney injury	41.2%	63.1%	<0.001
Incident dialysis	14.1%	17.9%	0.043
Acute encephalopathy	7.3%	14.1%	<0.001
Acute respiratory failure	13.9%	29.7%	<0.001
Acute respiratory distress syndrome	0.9%	1.3%	0.393
Sepsis	11.1%	15.4%	0.013
Acute heart failure	6.6%	17.9%	<0.001
Cardiogenic shock	1.8%	6.2%	<0.001
Acute cerebrovascular accident	6.3%	20.5%	<0.001
Comorbidities			
Collagen vascular disease	3.5%	3.9%	0.670
Hypertension	43.0%	53.1%	<0.001
Diabetes mellitus	16.9%	21.2%	0.031
Human immunodeficiency infection	1.7%	1.8%	0.840
Alcohol use disease	2.9%	3.6%	0.438
Chronic heart failure	8.2%	21.2%	<0.001
Chronic lung disease	10.1%	14.0%	0.020
Coagulation disease	14.2%	15.0%	0.653
Malignancy	5.5%	6.7%	0.306
Obesity	11.0%	9.3%	0.353
Cerebrovascular disease	3.2%	4.4%	0.235
Dyslipidemia	11.5%	16.4%	0.006
Smoking	5.6%	4.6%	0.493
Peripheral vascular disease	2.8%	5.7%	0.003
Coronary artery disease	6.2%	20.8%	<0.001
Chronic renal failure	15.4%	23.0%	<0.001
Mean number of procedures	4.8	5.9	<0.001
Early plasma exchange	52.8%	60%	0.005

**Figure 2:**
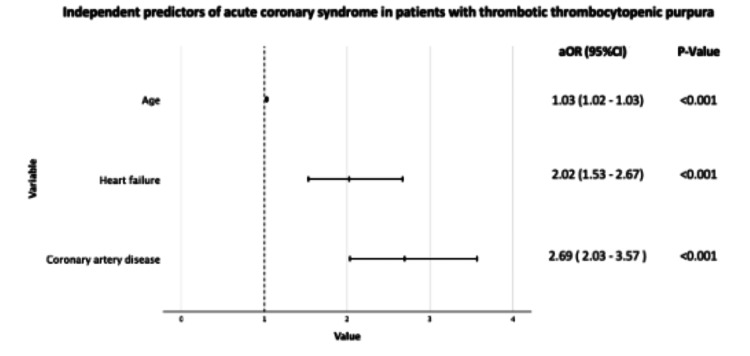
Factors associated with higher odds of ACS in patients admitted with TTP aOR, adjusted odds ratio; CI, confidence interval; ACS, acute coronary syndrome; TTP, thrombotic thrombocytopenic purpura.

Using stepwise logistic regression, we identified age (adjusted odds ratio [aOR], 1.03; 95% CI, 1.02-1.03; P<0.001), history of heart failure (aOR, 2.02; 95% CI, 1.53-2.67; P<0.001), and history of coronary artery disease (aOR, 2.69; 95% CI, 2.03-3.57; P<0.001) as independent predictors of ACS among patients hospitalized with TTP (Figure 2).

**Figure 3:**
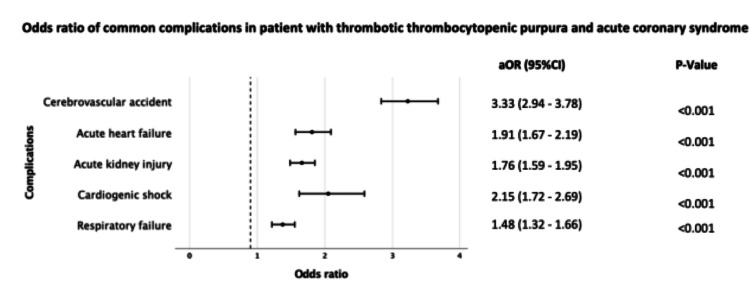
Complications that were more associated with patients with ACS as compared to those without ACS aOR, adjusted odds ratio; CI, confidence interval; ACS, acute coronary syndrome.

On regression analysis, certain diseases were more prevalent in the ACS group including acute cerebrovascular accidents (aOR, 3.33; 95% CI, 2.94-3.78; P<0.001), acute heart failure (aOR, 1.91; 95% CI, 1.67-2.19; P<0.001), acute kidney injury (aOR, 1.76; 95% CI, 1.59-1.95; P<0.001), cardiogenic shock (aOR, 2.15; 95% CI, 1.72-2.69; P<0.001), and respiratory failure (aOR, 1.48; 95% CI, 1.32-1.66; P<0.001) (Figure 3).

## Discussion

Using a large, nationally representative database of inpatient hospitalization, we report the predictors of ACC in a patient with TTP and the impact of ACC on the clinical course of TTP. The main findings of our study include the following: TTP patients had an in-hospital mortality rate of 9.1%. Older age, history of heart failure, and coronary artery disease are independent predictors of ACS in TTP patients. TTP patients with ACS had a three-fold higher in-hospital mortality, higher complication rates, and longer hospital stay.

Cardiac involvement in TTP patients includes ACS, tachyarrhythmia, heart failure, myocarditis, myocardial necrosis, cardiogenic shock, or sudden cardiac death [8-12]. Cardiac involvement was reported in the first case of TTP ever reported in the literature by Moschcowitz [1]. The patient had T wave inversion on ECG and the autopsy revealed thrombi in the terminal arterioles and capillaries in the heart. Cardiac thrombi/emboli were found to be the most common TTP-related finding on autopsy with acute myocardial infarction (AMI) and cardiac arrest being the most common immediate causes of death [13]. The usual cardiac risk factors such as diabetes, age, and hypertension were not found to be significantly increased in TTP patients with AMI [4].

Although elevated cardiac troponin level was reported in 59% of TTP patients on admission, TTP rarely presents with ACS and most of the patients are silent from the cardiac standpoint [14]. Therefore, some authors recommend using troponin as a prognostic indicator in patients with TTP. The discrepancy between the autopsy findings and the clinical data could be related to the fact that most cases were described in reports of a few selected patients. Also, many patients were reported before the availability of effective treatment for TTP and sensitive tests for cardiac involvement [8]. However, continuing case reports and studies suggest that cardiac involvement could be an important, underdiagnosed cause of morbidity and mortality in TTP patients. We found that TTP patients with ACS had more PLEX procedures compared to those without ACS. Our methods did not permit us to evaluate for causality. However, there is no evidence in the literature that PLEX is associated with adverse cardiac events. Furthermore, beneficial effects have been reported with PLEX in some cardiac diseases [15-17]. Further studies are required to characterize this relationship.

Many studies sought to identify factors associated with poor prognosis in patients with TTP. A large retrospective study has reported 11.1% in-hospital mortality in TTP patients. The study identified older age, AMI, acute renal failure, congestive heart failure, acute cerebrovascular disease, cancer, and sepsis as independent predictors of increased in-hospital mortality. AMI incidence was 5.7% in their cohort. Older age, smoking, known coronary artery disease, and congestive heart failure were independent predictors of myocardial infarction [18]. In concordance with this study, our analysis shows that age (OR, 1.03; 95% CI, 1.02-1.03), history of heart failure (OR, 2.02; 95% CI, 1.53-2.67), and history of coronary artery disease (OR, 2.69; 95% CI, 2.03-3.57) are independent predictors of ACS among patients hospitalized with TTP.

Multivariate analysis of in-hospital complications shows that acute kidney injury, acute respiratory failure, acute congestive heart failure, cerebrovascular accidents, and cardiogenic shock were significantly higher in ACS patients with adjusted OR varying from 1.76 to 3.33 (Figure 2). Previous studies on ACS patients have shown cardiogenic shock and acute heart failure as independent predictors of increased in-hospital mortality in patients with ACS [19,20]. These findings could explain the higher mortality rate in our cohort with ACS.

This report provides a much larger body of data on ACS in TTP patients. Our findings illustrate the impact of cardiac involvement on the prognosis of TTP patients. This should raise providers’ awareness of such complications and call for further studies to determine whether cardiac treatment would alter the course of the disease and improve the prognosis.

We acknowledge several limitations, some of which are inherent to any claims data analysis. Coding errors, unmeasured confounders, and under-reporting of comorbidities are potential limitations of using ICD codes. We followed the best practice as described by previous reports highlighting the use of claims data [21,22]. Long-term outcomes, as well as certain important clinical and laboratory parameters (such as ADAMTS13 activity), could not be evaluated as NIS reports only discharge-level information. Moreover, the database does not allow the identification of patients admitted with disease recurrence and does not provide data on all treatments administered during the hospital stay, which might contribute to potential bias.

## Conclusions

In this large retrospective study, patients with TTP who also developed ACS had significantly worse in-hospital outcomes compared to those who did not have ACS. We identified advanced age, history of heart failure, and coronary artery disease as independent predictors for ACS occurrence in TTP patients. Further studies are needed to identify the incidence and the impact of cardiac involvement in TTP. Prospective studies are needed to determine whether cardiac therapy would improve survival and long-term outcomes in TTP patients.

## Appendices

**Table 2:** ICD codes ICD-9-CM, International Classification of Disease Ninth Revision, Clinical Modification.

Diagnosis	ICD-9-CM code	ICD-10-CM code
Thrombotic microangiopathy	4466	M311
Acute coronary syndrome	4111, 41189, 41011, 41001, 41011, 41021, 41031, 41041, 41071, 41051, 41061, 41071, 41081, 41091	I200, I249, I2101, I2102, I2109, I2111, I2119, I2121, I2129, I213, I214, I220, I221, I222, I228, I229
Plasma exchange	Procedure codes: 9972, 9979	Procedure codes: 6A551Z3, 6A550Z3
